# Statin use in older people primary prevention on cardiovascular disease: an updated systematic review and meta-analysis

**DOI:** 10.31083/j.rcm2304114

**Published:** 2022-03-24

**Authors:** Hao Huang, Hechen Zhu, Ru Ya

**Affiliations:** ^1^Department of Critical Rehabilitation, Shanghai Third Rehabilitation Hospital, 200436 Shanghai, China; ^2^Department of Critical Care Medicine, Huashan Hospital, Fudan University, 200031 Shanghai, China

**Keywords:** statin, older people, cardiovascular disease, primary prevention, meta-analysis

## Abstract

**Background:**

Evidence on statin use for primary prevention of cardiovascular disease (CVD) in older people needs to be extended and updated, aiming to provide further guidance for clinical practice.

**Methods:**

PubMed, EMBASE, Cochrane Library and Web of Science were searched for eligible observational studies comparing statin use vs. no-statin use for primary prevention of CVD in older people (age ≥65 years). The primary outcomes were all-cause mortality, CVD mortality, coronary heart disease (CHD)/myocardial infraction (MI), stroke and total CV events. Risk estimates of each relevant outcome were synthesized as a hazard ratio (HR) with 95% confidence interval (95% CI) using in the random-effects model.

**Results:**

Twelve eligible observational studies (n = 1,627,434) were enrolled. The pooled results suggested that statin use was associated with a significantly decreased risk of all-cause mortality (HR: 0.54, 95% CI: 0.46–0.63), CVD mortality (HR: 0.51, 95% CI: 0.39–0.65), CHD/MI (HR: 0.83, 95% CI: 0.69–1.00), stroke (HR: 0.79, 95% CI: 0.68–0.92) and total CV events (HR: 0.75, 95% CI: 0.66–0.85). The association in all-cause mortality still remained obvious at higher ages (≥70 years old, HR: 0.56, 95% CI: 0.44–0.71; ≥75 years old, HR: 0.70, 95% CI: 0.60–0.80; ≥85 years old, HR: 0.85, 95% CI: 0.74–0.97), ≥20% (HR: 0.47, 95% CI: 0.35–0.62) and <20% diabetic populations (HR: 0.50, 95% CI: 0.40–0.64), and ≥50% (HR: 0.68, 95% CI: 0.59–0.79) and <50% hypertensive populations (HR: 0.38, 95% CI: 0.16–0.88).

**Conclusions:**

Statin use was related to a 46%, 49%, 17%, 21% and 25% risk reduction on all-cause mortality, CVD mortality, CHD/MI, stroke and total CV events in older patients, respectively. The significant association was also addressed in older patients and ≥75 years old individuals for CVD primary prevention.

## 1. Introduction

Cardiovascular disease (CVD) is a global burden, and more than 80% cases of mortality occur in older population (age ≥65 years) [1, 2, 3]. By 2030, the percentage of aging populations is projected to reach 1 billion (12% of the world populations) [4]. In Europe, almost 25% population will be aged ≥65 years at that time, which is higher than any other countries [4]. Accordingly, CVD prevention in older people is important and it has been regarded as agenda for global healthcare duties.

It is well-established that statin use is recommended for secondary prevention of CVD in older people as level A evidence, while considerable evidence for primary prevention is insufficient [5, 6]. Currently, statin therapy for high CVD risk people ≥75 years was supported by level B evidence and recommended as a class IIb priority by 2019 European Society of Cardiology (ESC)/European Atherosclerosis Society (EAS) guidlines [5, 6]. Different from the 2016 class IIa priority and level B evidence, the 2019 ESC/EAS guidelines advocated statins for primary prevention in older people who were no more than 75 years old as class I recommendation [7, 8]. An individual-level meta-analysis reported 39% of risk reduction in major vascular events for every 1 mmol/L drop in low-density lipoprotein cholesterol (LDL-C) with statins in older people from 65–70 years old without prior cardiovascular disease. The beneficial role of statin in more than 70 years old population was not obvious [9]. Overall, current evidence implied that the data are insufficient to draw conclusive results of the beneficial role of statin for primary prevention in older people.

Another meta-analysis reported reduced CVD risk in statin-use for secondary prevention over the primary prevention in older population, and the data are insufficient for the risk of onset diabetes [10]. The main limitations of former results include that they mainly focus on component outcomes (major vascular events) rather than specific outcomes (coronary heart disease (CHD), myocardial infraction (MI), stroke, etc.) [11]. Then, considering the strict inclusion criteria, older people were always omitted from clinical trials. Current results on the primary prevention for older populations were always from subgroup analyses, which is not enough [10, 12]. Worse more, evidence on this topic based on clinical trials was coupled with limited sample size of intended population in a short period of follow-up [12, 13]. To our point of view, the outcomes of interest like total CVD events were not reported in previous meta-analysis upon observational studies, which also lacked some key eligible studies [14]. Therefore, we could not have a comprehensive evaluation of the statin use for CVD primary prevention especially in older population. Observational studies in this scope may extend the current limited evidence with larger population and longer follow-up period. Herein, we conducted this meta-analysis based on observational studies to (1) investigate the CVD primary prevention via statin use in older population; (2) present the preventive association by age; (3) make updated clinical advice to high CVD risk population.

## 2. Methods 

According to the Cochrane Handbook and the Meta-analysis of Observational Studies in Epidemiology (MOSE) Guidelines Checklist and Preferred Reporting Items for Systematic Reviews and Meta-Analyses (PRISMA) guidelines (**Supplementary Table 1**) [15, 16], this study was designed. The protocol is consistent with a previous study [14], and has been registered on the INPLASY website (https://inplasy.com/) with a reference ID: INPLASY2021120045 (doi: 10.37766/inplasy2021.12.0045) (**Appendix File 1**).

### 2.1 Search strategy

We reviewed Pubmed, EMBASE, Cochrane Library and Web of Science for related literatures from the inception to Sep. 15th, 2021. We used a combination of relevant keywords and Medical Subject Headings (MeSH) terms, including “Aging”, “Aged”, “elderly”, “Statin”, “atorvastatin”, “cardiovascular disease”, “cardiovascular events”, “coronary heart disease”, “myocardial infarction”, “stroke” and “observational study”. Detailed search strategy is given in **Supplementary Table 2**. No restrictions were applied on language. Reference lists of the retrieved literature were also searched manually.

### 2.2 Selection criteria

All articles were screened in two-step methods. Two authors independently screened the studies’ titles and abstracts, then reviewed the full texts of potentially eligible studies. Any disagreements were resolved by another author who is exceptional in cardiology and evidence-based medicine from a discussion in a group panel.

The eligible criteria following PICOS principles are as follows.

#### 2.2.1 Populations 

Being limited to or including a subgroup of older people aged ≥65 years using statin for primary prevention. No further restrictions on additional individual-level characteristics (e.g., sex, ethnicity, and nation).

#### 2.2.2 Intervention/comparison

Statin (atorvastatin, fluvastatin, lovastatin, pitavastatin, pravastatin, rosuvastatin, or simvastatin) use vs. no statin use for primary prevention.

#### 2.2.3 Outcomes

Including at least one of the following outcomes: All-cause mortality, CVD mortality, CHD/MI, stroke or total CV events.

#### 2.2.4 Study design: observational study 

Only the most informative studies with longer follow-up (no less than one year considering the limited life expectancy of older people) could be included to avoid duplication. Clinical trials, reviews, case reports, conference abstracts, experimental studies, and studies without essential data were excluded.

### 2.3 Data extraction and outcome of interest

Two independent authors performed data extraction following a prespecified protocol from eligible studies. The extracted information included characteristics of the eligible studies (year of publication, first author, study design, study location, follow-up period, etc.), characteristics of the populations (median age and sample size), and the characteristics of the program (systematic exposure, outcomes of endpoints, adjusted confounders, registration information, etc.). All risk estimates were evaluated in fully adjusted models. Intention-to-treat principles (ITT) were applied if available, and the primary authors would be contacted if there were missing data. However, analyses would still have been taken without these data if no response was received.

The primary outcomes included risk of all-cause mortality, CVD mortality, CHD/MI, stroke and total CV events, because they had most clinical significance and abundant useful data. Secondary outcomes included risk estimate on no diabetes mellitus (NODM) and cancer incidence. Detailed definitions about outcomes of interest have been summarized in **Supplementary material 1**. The data regarding older people who survived from the first age to a new age were reported by independent cohorts, respectively, and then the data could be deemed as being achieved from two different cohorts. The methods to avoid duplication have been addressed in the selection criteria part.

### 2.4 Quality assessment

To evaluate the quality of included studies, we applied the Newcastle-Ottawa Scale (NOS) as previously, which has been validated to assess the quality of nonrandomized controlled trials in meta-analyses [17]. As for a 0–10 scale, each study was categorized as low (0–5), medium (6–7), and high (8–10) quality. Two authors performed a quality assessment on all of the included studies based on the method. In case of any disagreements, there would be a discussion between the two authors.

Afterwards, we used the Risk of Bias in Non-randomized Studies of Interventions (ROBINS-I) tool to make further risk estimates on the included studies [18]. This tool displays 7 items and classifies the risks of bias into low, moderate, serious, critical and unclear risks. The process was completed by two independent authors and there would be a discussion in case of any disagreements.

### 2.5 Evidence grade evaluation

In this case, we applied the Grading of Recommendation Assessment, Development and Evaluation (GRADE) approach to identify the level rating of each outcome of interest as very low, low, moderate, or high quality [19]. The rating system follows 5 items: risk of bias, imprecision, inconsistency, indirectness, publication bias, large effect size, dose-response gradient and all residual confounding reducing an effect size [20, 21]. If there was one “serious” item, the evidence level could have been regarded as “low”; and if there was one “very serious”, the evidence level been “very low”.

### 2.6 Statistical analysis

Multivariable hazard ratio (HR) and the corresponding 95% confidence intervals (95% CIs) for outcome of interests obtained from Cox-Hazard regression analysis were mainly estimated with DerSimonian-Laird (D-L) random effects model, because the assumptions involved accounted for the presence of within-study and between-study heterogeneity. In order to provide the most comprehensive results, both fixed- and random-effects models results were shown in the forest plots. The adjusted relative risk (RR) and odd ratio (OR) in primary studies were approximately considered as HR. Fully adjusted HRs and standard errors (SEs) originating from the correspondence 95% CIs were logarithmically transformed to stabilized variance, and the distribution was normalized. Between-study heterogeneity was determined with the Cochran Q chi-square test and *$I^{2}$*. An *$I^{2}$* >50% or a *p* value for the Q test <0.1 was deemed as revealing significant heterogeneity [22].

In addition, a sensitivity analysis was performed by moving one study each turn to try to elaborate the causes of the heterogeneity. We would also conduct post subgroup analyses to ascertain the influence of other design and individual factors as follows: different categories on age, region, diabetic characteristics, hypertension status, study follow-up period and study design.

Publication bias was investigated by Egger’s linear regression tests at *p *< 0.10 of significant bias and visualized by trim-and-filling funnel plots [23]. All analyses were performed using R software version 3.5.3 (www.r-project.org) with publicly available “meta” package; two-sided *p *< 0.05 was statistically significant, except specified one.

## 3. Results

### 3.1 Study selection and characteristics of the included studies

**Fig. 1.**
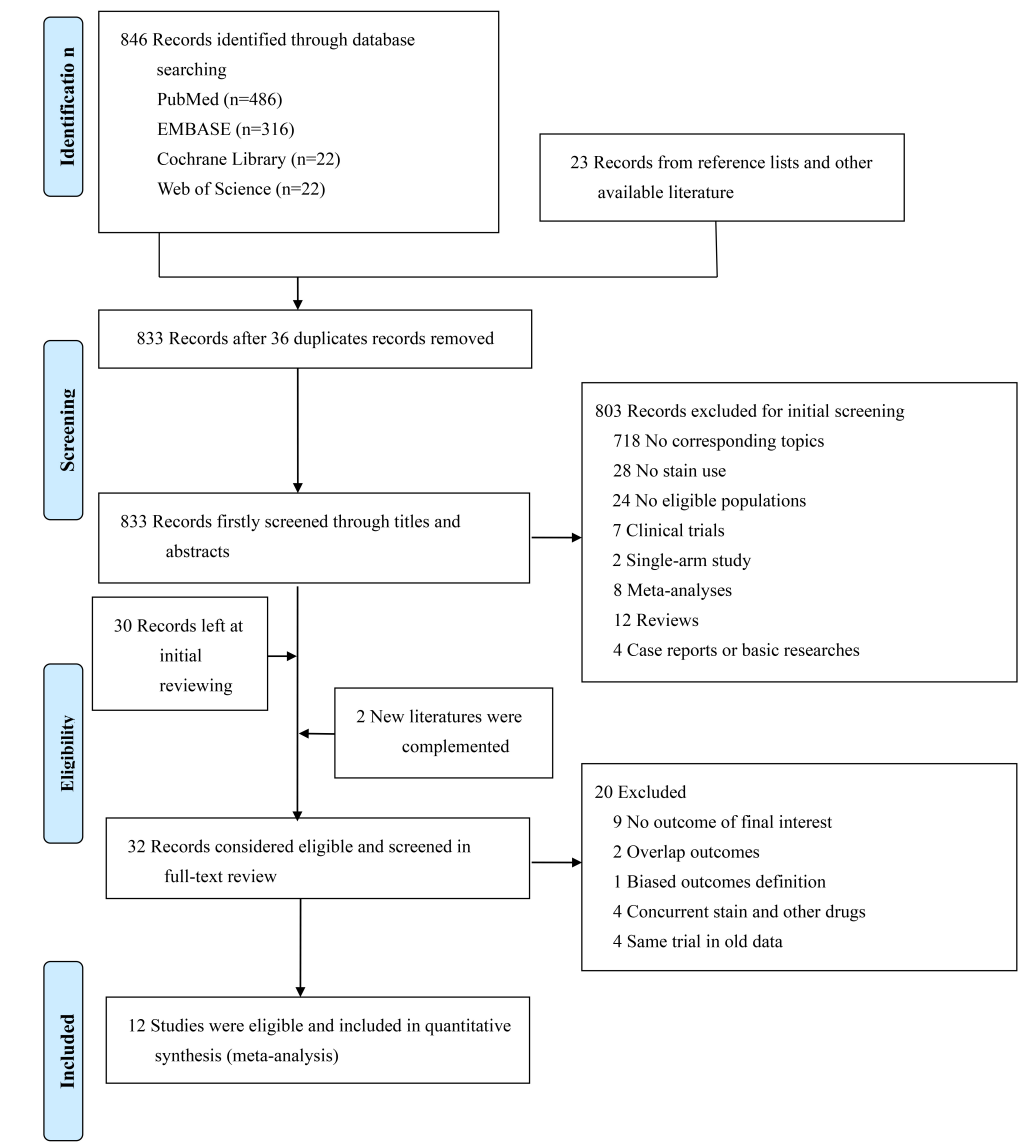
**The flow chart for study screening and selection**.

Among 869 studies (846 from the main searched databases (PubMed = 486, EMBASE = 316, Cochrane Library = 22, Web of Science = 22) and 23 from other related literature), 803 studies were excluded after initial screening, and 20 studies were excluded after full consideration due to no required outcomes of final interest, overlapped outcomes, different types of statin plus other drugs, biased outcomes definition, etc. (Fig. 1).

A total of 12 observational studies incorporating 1,627,434 population were eligible for this analysis [24, 25, 26, 27, 28, 29, 30, 31, 32, 33, 34, 35]. Detailed characteristics were summarized in Table 1 (Ref. [24, 25, 26, 27, 28, 29, 30, 31, 32, 33, 34, 35]). All eligible studies involved ≥65 years individuals, three studies only involved ≥70 years individuals [27, 34, 35] and 5 studies only involved ≥75 years individuals [28, 29, 30, 31, 32]. One study was conducted on all males [27], and one study was performed on NODM individuals [29]. There were 11 studies reported all-cause mortality [24, 26, 27, 28, 29, 30, 31, 32, 33, 34, 35] outcomes, 5 studies reported CVD mortality [24, 31, 32, 33, 34], 8 studies reported CHD/MI [24, 25, 27, 28, 30, 31, 32, 34] outcomes, 8 studies reported stroke [24, 25, 27, 28, 30, 31, 32, 34] outcomes, 8 studies reported total CV events [24, 25, 27, 28, 30, 32, 34, 35], three studies provided DM [28, 31, 35] endpoint, and 2 studies revealed cancer incidence [28, 31]. Three studies were with prospective study design [24, 25, 27], 7 studies belonged to retrospective study [26, 28, 29, 31, 32, 34, 35], 1 study was nest case-control study [30], and 1 study was case-control study [33]. Three studies were conducted in the USA [24, 27, 32], 1 study was carried out in the USA and Asutralia [34], 5 studies were in Europe (including UK) [25, 26, 28, 29, 33], and 3 studies were in Asia [30, 31, 35]. The mean follow-up period was 7.49 years. The confounders of adjustment in each study have been listed in **Supplementary Table 3**.

**Table 1.** **Characteristics of each included studies**.

Study	Population	Follow-up period (y)	Groups	Mean Age (y)	Female (n/%)	BMI (kg/$m^{2}$)	TC† (mmol/L)	LDL-C† (mmol/L)	HDL-C† (mmol/L)	TG‡ (mmol/L)	Smoking status* (%)	Alcoholic Status* (%)	DM (%)	HTN (%)	Outcomes	Study Design
Lemaitre *et al*. [24], 2002 (USA)	≥65 years people without prior CVD	7.3	Stain used group (n = 251)	71.1 (4.6)	172 (68.5)	49.4	5.83 (1.1)	3.70 (1.1)	1.39 (0.4)	1.78 (1.0)	9.6	49.4	21.9	48.20%	①, ②, ③, ④, ⑤	Prospective cohort study
			Stain recommended group (n = 717)	72.7 (5.6)	478 (66.7)	27.5 (4.5)	6.70 (0.9)	4.59 (0.7)	1.31 (0.3)	1.76 (0.7)	14.6	45.3	20.5	48.1		
			Diet recommended group (n =946)	72.5 (5.3)	600 (63.4)	27.2 (5.0)	5.92 (0.7)	3.82 (0.5)	1.37 (0.4)	1.63 (0.7)	13.9	48.9	20	43.7		
Alpérovitch *et al*. [25], 2015 (France)	≥65 years people without prior CVD	9.1	Stain used group (n = 1007)	73.1 (4.6)	683 (67.8)	25.8 (4.0)	5.68 (0.9)	3.40 (0.9)	1.64 (0.4)	1.27 (0.84–1.93)§	34.6	82.9	10.9	79.7	③, ④, ⑤	Prospective cohort study
			No stain use group (n = 5436)	74.1 (5.6)	3368 (62.0)	25.4 (4.0)	5.97 (1.0)	3.78 (0.9)	1.63 (0.4)	1.14 (0.76 to 1.70)§	37.6	82.5	7.2	74.5		
Gitsels *et al*. [26], 2016 (UK)	65, 70, 75 years people without prior CVD stratified by QRISK2 Score	16–24	Stain used group in QRISK <10% (n = 883)	65	833 (100)	26.0 (4.0)	NA	NA	NA	NA	10	NA	0	NA	①	Retrospective study
			No stain used group in QRISK <10% (n = 39866)	65	39866 (100)	26.0 (4.0)	NA	NA	NA	NA	13	NA	0	NA		
			Stain used group in QRISK <10% (n = 3)	70	3 (100)	28.0 (6.0)	NA	NA	NA	NA	0	75.6	0	NA		
			No stain used group in QRISK <10% (n = 322)	70	322 (100)	25.0 (4.0)	NA	NA	NA	NA	23		0	NA		
			Stain used group in QRISK 10–19% (n = 6438)	65	4381 (68)	28.0 (5.0)	NA	NA	NA	NA	34	NA	7	NA		
			No stain used group in QRISK 10–19% (n = 116240)	65	54094 (47)	26.0 (4.0)	NA	NA	NA	NA	41	NA	1	NA		
			Stain used group in QRISK 10–19% (n = 10822)	70	9928 (92)	27.0 (5.0)	NA	NA	NA	NA	21	NA	0	NA		
			No stain used group in QRISK 10–19% (n = 108703)	70	93010 (86)	26.0 (5.0)	NA	NA	NA	NA	22	NA	0	NA		
			Stain used group in QRISK 10–19% (n = 661)	75	661 (100)	26.0 (4.0)	NA	NA	NA	NA	5	NA	0	NA		
			No stain used group in QRISK 10–19% (n = 13685)	75	13684 (100)	25.0 (4.0)	NA	NA	NA	NA	6	NA	0	NA		
			Stain used group in QRISK ≥20% (n = 5259)	65	1742 (33)	29.0 (5.0)	NA	NA	NA	NA	64	NA	59	NA		
			No stain used group in QRISK ≥20% (n = 29170)	65	4532 (16)	27.0 (5.0)	NA	NA	NA	NA	76	NA	22	NA		
			Stain used group in QRISK ≥20% (n = 25559)	70	9570 (37)	29.0 (5.0)	NA	NA	NA	NA	56	NA	39	NA		
			No stain used group in QRISK ≥20% (n = 98900)	70	23626 (24)	26.0 (4.0)	NA	NA	NA	NA	59	NA	8	NA		
			Stain used group in QRISK ≥20% (n = 34743)	75	19566 (56)	28.0 (5.0)	NA	NA	NA	NA	44	NA	29	NA		
			No stain used group in QRISK ≥20% (n = 142521)	75	78799 (55)	26.0 (4.0)	NA	NA	NA	NA	41	NA	5	NA		
Orkaby *et al*. [27], 2017 (USA)	≥70 years people without prior CVD	7	Stain used group (n = 1130)	76.0 (4.5)	0 (0)	25.6 (3.1)	NA	NA	NA	NA	51.8	85.1	13	73.8	①, ③, ④, ⑤	Prospective cohort study
			No stain use group (n = 1130)	76.0 (4.6)	0 (0)	25.6 (3.2)	NA	NA	NA	NA	53.8	85.9	13.1	75.3		
Ramos *et al*. [28], 2018 (Spain)	≥75 years people without prior CVD	5.6	Stain used in 75–84 years, no DM group (n = 4802)	78.8 (2.7)	3126 (65.1)	28.6 (4.6)	6.1 (1.1)	3.9 (1.0)	1.5 (0.4)	1.4 (0.7)	13.5	NA	NA	65.7	①, ③, ④, ⑤, ⑥, ⑦	Retrospective study
			No stain used in 75–84 years, no DM group (n = 27114)	79.1 (2.8)	17028 (62.8)	28.4 (4.6)	5.4 (0.9)	3.3 (0.7)	1.5 (0.4)	1.2 (0.5)	12.4	NA	NA	57.3		
			Stain used in ≥85 years, no DM group (n = 743)	88.5 (3.2)	519 (69.8)	27.1 (4.3)	5.9 (1.2)	3.7 (1.0)	1.5 (0.4)	1.4 (0.6)	7.8	NA	NA	66.8		
			No stain used in ≥85 years, no DM group (n = 6325)	88.6 (3.2)	4415 (69.8)	27.6 (4.5)	5.2 (0.9)	3.1 (0.8)	1.6 (0.4)	1.2 (0.5)	6.7	NA	NA	58.7		
			Stain used in 75–84 years, DM group (n = 1756)	78.8 (2.6)	1076 (61.3)	29.7 (4.7)	5.8 (1.1)	3.7 (0.9)	1.4 (0.4)	1.7 (0.8)	15.4	NA	NA	78.4		
			No stain used in 75–84 years, DM group (n = 4885)	79.2 (2.8)	2833 (58)	29.4 (4.8)	5.0 (0.8)	3.0 (0.7)	1.4 (0.4)	1.4 (0.7)	14.7	NA	NA	75.1		
			Stain used in ≥85 years, DM group (n = 201)	88.2 (2.8)	135 (67.2)	29.7 (4.7)	5.8 (1.1)	3.7 (0.9)	1.4 (0.4)	1.7 (0.8)	15.4	NA	NA	78.4		
			No stain used in ≥ 85 years, DM group (n = 1038)	88.2 (2.7)	706 (68)	29.4 (4.8)	5.0 (0.8)	3.0 (0.7)	1.4 (0.4)	1.4 (0.7)	14.7	NA	NA	75.1		
Bezin *et al*. [29], 2019 (France)	≥75 years people without prior CVD	4.7	Primary prevention without modifiable risk factors (n = 752)	78 (76–81)	540 (71.8)	NA	NA	NA	NA	NA	NA	NA	0	NA	①	Retrospective study
Jun *et al*. [30], 2019 (South Korea)	≥75 years people without prior CVD	NA	Cases (n = 11017)	83.7 (3.2)	6966 (66.4)	NA	NA	NA	NA	NA	NA	NA	14.7	44.2	①, ③, ④, ⑤	Nested case-control study
			Controls (n = 55085)	83.7 (3.2)	34830 (63.2)	NA	NA	NA	NA	NA	NA	NA	11.5	49.9		
Kim *et al*. [31], 2019 (South Korea)	≥75 years people without prior CVD	5.2	Stain used group (n = 639)	78 (76–80)	413 (64.6)	23.4 (22.2–25.8)§	4.46 (3.78–5.18)§	2.77 (2.20–3.45)§	1.17 (1.01–1.40)§	1.27 (0.94–1.73)§	NA	NA	32.6	95.6	①, ②, ③, ④, ⑥, ⑦	Retrospective study
			No stain use group (n = 639)	78 (76–80)	392 (61.3)	23.3 (22.0–25.6)§	4.40 (3.86–5.16)§	2.77 (2.20–3.34)§	1.19 (0.98–1.42)§	1.23 (0.91–1.74)§	NA	NA	30.8	95.9		
Orkaby *et al*. [32], 2020 (USA)	≥75 years people without prior CVD	6.8	Stain used group (n = 57178)	81.2 (3.6)	1544 (2.7)	27.5 (4.3)	NA	NA	NA	NA	70.9	NA	27	80.4	①, ②, ③, ④, ⑤	Retrospective study
			No stain use group (n = 326981)	80.7 (4.0)	8828 (2.7)	26.7 (4.4)	NA	NA	NA	NA	79.2	NA	13.1	66.2		
Rea *et al*. [33], 2020 (Italy)	≥65 years people without prior CVD	7	Good clinical frailty group (n = 82782)	73.0 (6.0)	49249 (59.5)	NA	NA	NA	NA	NA	NA	NA	13.5	NA	①, ②	Case-control study
			Intermediate clinical frailty group (n = 175771)	74.0 (6.0)	96138 (54.7)	NA	NA	NA	NA	NA	NA	NA	10.4	NA		
			Poor clinical frailty group (n = 170483)	76.0 (7.0)	81331 (47.7)	NA	NA	NA	NA	NA	NA	NA	NA	NA		
			Very poor clinical frailty (n = 31424)	76.0 (6.0)	12801 (40.7)	NA	NA	NA	NA	NA	NA	NA	NA	NA		
Zhou *et al*. [34], 2020 (Australia and USA)	≥70 years people without prior CVD	4.7	Stain used group (n = 5629)	74.2 (71.8–77.7)	3413 (60.6)	NA	NA	NA	NA	NA	45.4	75.6	19.6	82.4	①, ②, ③, ④, ⑤	Retrospective study
			No stain use group (n = 12467)	74.2 (71.8–77.9)	6727 (54.0)	NA	NA	NA	NA	NA	44	78.3	6.1	70.8		
Lavie *et al*. [35], 2021 (Israel)	≥70 years people without prior CVD	5	≥70 years populations (n = 5970)	76.9 (5.9)	3699 (62)	NA	NA	NA	NA	NA	26.5	NA	NA	NA	①, ⑤, ⑥	Retrospective study

Continuous data was presented as mean (standard deviation, SD); Dichotomous data was presented as percentage. *Percentage of smoking and alcohol use was calculated by current + past use. †In TC, LDL-C, HDL-C, 1 mmol/L = 38.6 mg/dL. ‡In TG, 1 mmol/L = 86.8 mg/dL. §Data was presented as median (interquartile range). Abbreviations: BMI, body mass index; TC, total cholesterol; LDL-C, low density lipoprotein-cholesterol; HDL-C, low density lipoprotein-cholesterol; TG, triglyceride; DM, diabetes mellitus; HTN, hypertension; CVD, cardiovascular disease; NA, not available. Outcomes: ①, All-cause mortality; ②, CVD mortality; ③, Coronary heart disease (CHD)/Myocardial infraction (MI); ④, Stroke; ⑤, Total CV events; ⑥, New onsets on DM; ⑦, New onsets on cancer.

Regarding the study quality by NOS, the average NOS score was 6.67. Among all 12 studies, there were 2 low quality studies [29, 30], 6 medium quality studies [26, 27, 31, 33, 34, 35], and 4 high quality studies [24, 25, 28, 32]. Limited by the life expectancy, most studies were lack of adequate follow-up periods (**Supplementary Table 4**). With ROBINS-I tool, there were 5 studies [28, 29, 31, 32, 33] of moderate overall bias and the others [24, 25, 26, 27, 30, 34, 35] were of serious overall bias (**Supplementary Table 5**).

### 3.2 Analysis on primary outcomes

#### 3.2.1 All-cause mortality

**Fig. 2.**
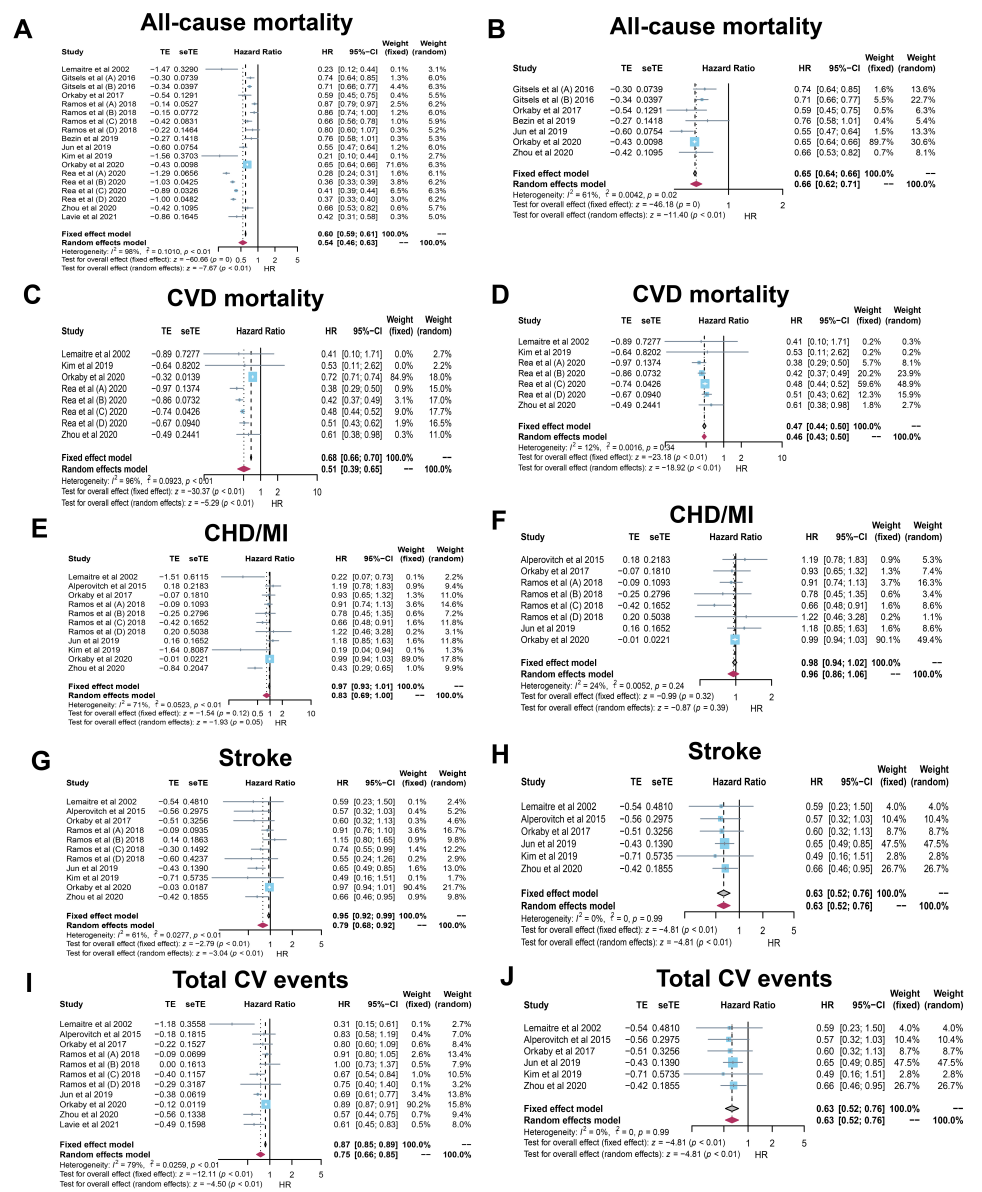
**Forrest plots for the primary outcomes**. CVD, cardiovascular disease; CHD/MI, coronary heart disease/myocardial infraction; HR, hazard ratio.

Eleven studies on all-cause mortality showed that the risk was reduced by 46% (HR: 0.54, 95% CI: 0.46–0.63; *p *< 0.01) with significant heterogeneity (*$I^{2}$* = 98%; *p *< 0.01) (Fig. 2A). After removing 5 heterogeneous studies [24, 28, 31, 33, 35], the HR turned to (HR: 0.66, 95% CI: 0.62–0.71; *p *< 0.01) with a little heterogeneity (*$I^{2}$* = 61%; *p* = 0.02) (Fig. 2B). The reduced risk changed into 34%, but it was still significant.

#### 3.2.2 CVD mortality

Five studies on CVD mortality displayed that the risk was reduced by 49% (HR: 0.51, 95% CI: 0.39–0.65; *p *< 0.01) with significant heterogeneity (*$I^{2}$* = 96%; *p *< 0.01) (Fig. 2C). After removing 1 heterogeneous study [32], the HR turned to (HR: 0.46, 95% CI: 0.43–0.50; *p *< 0.01) with little heterogeneity (*$I^{2}$* = 12%; *p* = 0.34) (Fig. 2D). The reduced risk changed into 54%, and the negative association was further confirmed.

#### 3.2.3 CHD/MI

Eight studies on CHD/MI demonstrated that the risk was reduced by 17% (HR: 0.83, 95% CI: 0.69–1.00; *p* = 0.05) with significant heterogeneity (*$I^{2}$* = 71%; *p *< 0.01) (Fig. 2E). After 3 heterogenous studies were removed [24, 31, 34], the HR turned to (HR: 0.96, 95% CI: 0.84–1.06; *p* = 0.39) with little heterogeneity (*$I^{2}$* = 24%; *p* = 0.24) (Fig. 2F). The CHD/MI results turned to be insignificant, which suggested that the significant association between statin use and CHD/MI was not robust and still required further related studies in the future.

#### 3.2.4 Stroke

Eight studies on stroke revealed that the risk was decreased by 21% (HR: 0.79, 95% CI: 0.68–0.92; *p *< 0.01) with significant heterogeneity (*$I^{2}$* = 61%; *p *< 0.01) (Fig. 2G). By omitting 2 studies of great heterogeneity [28, 32], we found that the HR was 0.63 (95% CI: 0.52–0.76; *p *< 0.01) with no heterogeneity (*$I^{2}$* = 0%; *p* = 0.99) (Fig. 2H). Reduced risk changed to 47% and became more robust.

#### 3.2.5 Total CV events

As for Total CV events, there were 8 relevant studies. The risk was reduced by 25% (HR: 0.75, 95% CI: 0.66–0.85; *p *< 0.01) with significant heterogeneity found (*$I^{2}$* = 79%; *p *< 0.01) (Fig. 2I). After 3 heterogeneous studies were removed [24, 28, 32], we discovered that the HR was (HR: 0.68, 95% CI: 0.61–0.76; *p *< 0.01) with little heterogeneity (*$I^{2}$* = 12%; *p* = 0.34) (Fig. 2J). The reduced risk changed to 32% and seemed to be more confirming.

### 3.3 Analysis on secondary outcomes

#### 3.3.1 DM incidence

**Fig. 3.**
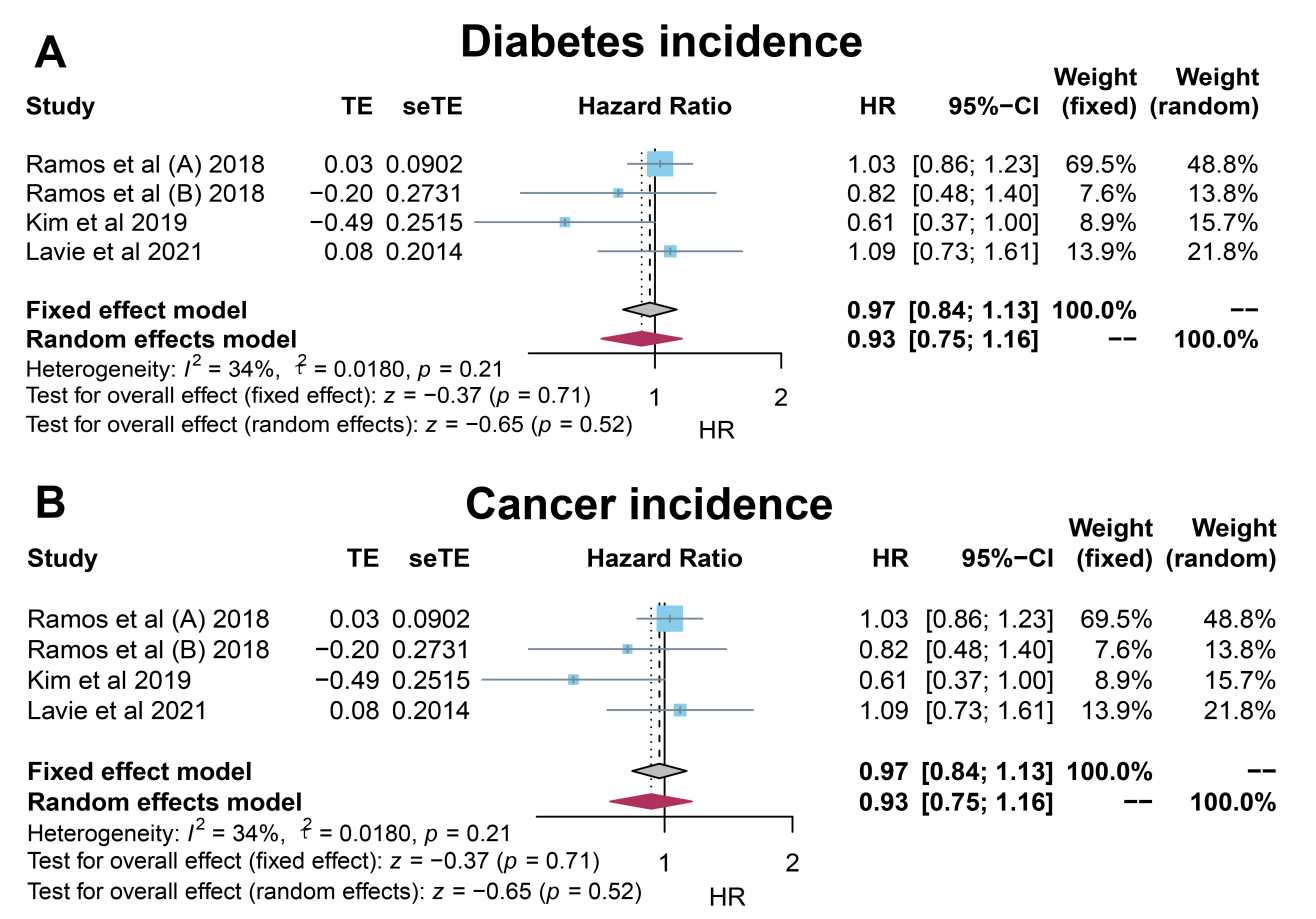
**Forrest plots for the secondary outcomes**. HR, hazard ratio.

Three studies on new onset of DM indicated that statin use had no significant association with primary prevention on DM incidence (HR: 0.93, 95% CI: 0.75–1.16; *p* = 0.52). There was no significant heterogeneity (*$I^{2}$* = 34%; *p* = 0.21) (Fig. 3A).

#### 3.3.2 Cancer incidence

Two studies on cancer incidence illustrated that statin use had no significant association with primary prevention on cancer incidence (HR: 0.98, 95% CI: 0.88–1.08; *p* = 0.66). There was no significant heterogeneity (*$I^{2}$* = 0%; *p* = 0.44) (Fig. 3B).

#### 3.3.3 Subgroup analysis

In the subgroup analyses, the reduced risk with statin use on all-cause mortality primary prevention kept robust across all subgroups, including ≥65 years (HR: 0.42, 95% CI: 0.32–0.55), ≥70 years (HR: 0.56, 95% CI: 0.44–0.71), ≥75 years (HR: 0.70, 95% CI: 0.60–0.80), and ≥85 years (HR: 0.85, 95% CI: 0.74–0.97) individuals; North America (HR: 0.59, 95% CI: 0.48–0.72), Europe (HR: 0.57, 95% CI: 0.45–0.73), and Asia (HR: 0.42, 95% CI: 0.28–0.62) individuals; ≥20% (HR: 0.47, 95% CI: 0.35–0.65) and <20% diabetes status (HR: 0.50, 95% CI: 0.40–0.64); ≥50% (HR: 0.68, 95% CI: 0.59–0.79) and <50% (HR: 0.38, 95% CI: 0.16–0.88) hypertension proportion individuals; ≥7 (HR: 0.44, 95% CI: 0.34–0.56) and <7 years follow-up period (HR: 0.68, 95% CI: 0.59–0.79) studies; prospective (HR: 0.38, 95% CI: 0.15–0.96), retrospective (HR: 0.70, 95% CI: 0.63–0.77), nested case–control (HR: 0.55, 95% CI: 0.47–0.64) and case control study (HR: 0.35, 95% CI: 0.31–0.41) (Table 2). For stroke and total CV events, most subgroup results were consistent with the final pooled results; for CHD/MI, the results of subgroups seemed to be inconsistent, which suggested that we still required more relevant studies to consolidate these findings (Table 2). Different from all-cause mortality, the association between statin use and CHD/MI, stroke, total CV events in ≥85 years individuals was not obvious. Overall, these subgroup analyses insisted the significantly reduced risk of all-cause mortality across the subgroups, and statin use could be potentially recommended for high diabetic proportion, high hypertensive proportion and ≥75 years old individuals.

**Table 2.** **Subgroup results on all-cause mortality, CHD/MI, stroke, total CV events**.

Subgroup		No. of studies	HR (95% CI) on fixed-effects model	HR (95% CI) on random-effects model	Final pooled HR (95% CI) on fixed-effects model	Final pooled HR (95% CI) on random-effects model	$I^{2}$, *p* for heterogeneity
All-cause mortality							
Age (y)		11					
	≥65	3	0.44 (0.43–0.46)	0.42 (0.32–0.55)	0.66 (0.64–0.67)	0.70 (0.60–0.80)	95%, *p *< 0.01
	≥70	3	0.58 (0.50–0.67)	0.56 (0.44–0.71)			61%, *p* = 0.08
	≥75	5	0.66 (0.64–0.67)	0.70 (0.60–0.80)			89%, *p *< 0.01
	≥85*	1	0.85 (0.74–0.97)	0.85 (0.74–0.97)			NA
Region		11					
	North America	4	0.65 (0.63–0.66)	0.59 (0.48–0.72)	0.51 (0.45–0.58)	0.42 (0.28–0.62)	72%, *p* = 0.01
	Europe	4	0.50 (0.49–0.52)	0.57 (0.45–0.73)			98%, *p *< 0.01
	Asia	3	0.51 (0.45–0.58)	0.42 (0.28–0.62)			75%, *p* = 0.02
Diabetes proportion (%)		9					
	≥20	4	0.64 (0.63–0.66)	0.47 (0.35–0.65)	0.60 (0.59–0.61)	0.48 (0.40–0.59)	87%, *p *< 0.01
	<20	5	0.46 (0.44–0.47)	0.50 (0.40–0.64)			97%, *p *< 0.01
Hypertension proportion (%)		6					
	≥50	4	0.65 (0.64–0.66)	0.68 (0.59–0.79)	0.65 (0.64–0.66)	0.63 (0.55–0.73)	85%, *p *< 0.01
	<50	2	0.53 (0.46–0.61)	0.38 (0.16–0.88)			80%, *p *< 0.01
Follow-up (y)		10					
	≥7	4	0.45 (0.43–0.46)	0.44 (0.34–0.56)	0.60 (0.60–0.61)	0.54 (0.46–0.64)	98%, *p *< 0.01
	<7	6	0.66 (0.64–0.67)	0.68 (0.59–0.79)			87%, *p *< 0.01
Study design		11					
	Prospective study	2	0.52 (0.41–0.65)	0.38 (0.15–0.96)	0.60 (0.59–0.61)	0.54 (0.46–0.63)	86%, *p *< 0.01
	Retrospective study	7	0.66 (0.65–0.67)	0.70 (0.63–0.77)			86%, *p *< 0.01
	Nested case-control study	1	0.55 (0.47–0.64)	0.55 (0.47–0.64)			NA
	Case control study	1	0.37 (0.36–0.39)	0.35 (0.31–0.41)			90%, *p *< 0.01†
CHD/MI							
Age (y)		8					
	≥65	2	0.99 (0.66–1.47)	0.56 (0.11–2.93)	0.97 (0.93–1.01)	0.83 (0.69–1.00)	85%, *p *< 0.01
	≥70	2	0.66 (0.51–0.87)	0.64 (0.30–1.35)			87%, *p *< 0.01
	≥75	4	0.98 (0.94–1.02)	0.91 (0.77–1.07)			51%, *p* = 0.06
	≥85*	1	0.87 (0.54–1.40)	0.87 (0.54–1.40)			NA
Region		8					
	North America	4	0.97 (0.93–1.02)	0.66 (0.41–1.06)	0.97 (0.93–1.01)	0.83 (0.69–1.00)	86%, *p *< 0.01
	Europe	2	0.87 (0.75–1.02)	0.87 (0.71–1.08)			30%, *p* = 0.22
	Asia	2	1.10 (0.75–1.02)	0.57 (0.10–3.23)			79%, *p* = 0.03
Diabetes proportion (%)		7					
	≥20	4	0.99 (0.94–1.03)	0.82 (0.53–1.25)	0.98 (0.94–1.02)	0.80 (0.59–1.08)	73%, *p* = 0.01
	<20	3	0.78 (0.62–0.98)	0.78 (0.44–1.40)			85%, *p *< 0.01
Hypertension proportion (%)		8					
	≥50	6	0.97 (0.93–1.01)	0.82 (0.67–1.00)	0.97 (0.93–1.01)	0.83 (0.69–1.00)	71%, *p *< 0.01
	<50	2	1.05 (0.77–1.44)	0.56 (0.11–2.89)			86%, *p *< 0.01
Follow-up (y)		7					
	≥7	3	0.95 (0.73–1.24)	0.81 (0.46–1.45)	0.97 (0.93–1.01)	0.79 (0.64–0.97)	71%, *p* = 0.03
	<7	4	0.97 (0.93–1.01)	0.75 (0.58–0.97)			77%, *p *< 0.01
Study design		8					
	Prospective study	3	0.95 (0.73–1.24)	0.81 (0.46–1.45)	0.97 (0.93–1.01)	0.83 (0.69–1.00)	71%, *p* = 0.03
	Retrospective study	4	0.97 (0.93–1.01)	0.75 (0.58–0.97)			77%, *p *< 0.01
	Nested case-control study	1	1.18 (0.85–1.63)	1.18 (0.85–1.63)			NA
Stroke							
Age (y)							
	≥65	2	0.58 (0.35–0.95)	0.58 (0.35–0.95)	0.95 (0.92–0.99)	0.79 (0.68–0.92)	0%, *p* = 0.97
	≥70	2	0.64 (0.47–0.88)	0.64 (0.47–0.88)			0%, *p* = 0.79
	≥75	4	0.96 (0.93–0.99)	0.85 (0.73–1.00)			62%, *p* = 0.01
	≥85*	1	1.02 (0.73–1.42)	0.88 (0.44–1.76)			NA
Region							
	North America	4	0.97 (0.93–1.00)	0.77 (0.56–1.05)	0.95 (0.92–0.99)	0.79 (0.68–0.92)	60%, *p* = 0.06
	Europe	2	0.87 (0.76–0.99)	0.84 (0.68–1.03)			42%, *p* = 0.14
	Asia	2	0.64 (0.49–0.83)	0.64 (0.49–0.83)			0%, *p* = 0.64
Diabetes proportion (%)							
	≥20	4	0.96 (0.93–1.00)	0.76 (0.53–1.07)	0.96 (0.92–0.99)	0.70 (0.53–0.91)	72%, *p* = 0.01
	<20	3	0.63 (0.48–0.83)	0.63 (0.48–0.83)			0%, *p* = 0.91
Hypertension proportion (%)							
	≥50	6	0.96 (0.93–0.99)	0.83 (0.72–0.97)	0.95 (0.92–0.99)	0.79 (0.68–0.92)	53%, *p* = 0.03
	<50	2	0.64 (0.49–0.83)	0.64 (0.49–0.83)			0%, *p* = 0.84
Follow-up (y)							
	≥7	3	0.58 (0.40–0.86)	0.58 (0.40–0.86)	0.96 (0.92–0.99)	0.83 (0.71–0.96)	0%, *p* = 1.00
	<7	4	0.96 (0.93–1.00)	0.88 (0.76–1.01)			49%, *p* = 0.07
Study design							
	Prospective study	3	0.58 (0.40–0.86)	0.58 (0.40–0.86)	0.95 (0.92–0.99)	0.79 (0.68–0.92)	0%, *p* = 1.00
	Retrospective study	4	0.96 (0.93–1.00)	0.88 (0.76–1.01)			49%, *p* = 0.07
	Nested case-control study	1	0.65 (0.49–0.85)	0.65 (0.49–0.85)			NA
Total CV events							
Age (y)		8					
	≥65	2	0.68 (0.49–0.93)	0.53 (0.20–1.40)	0.87 (0.85–0.89)	0.75 (0.66–0.85)	84%, *p* = 0.01
	≥70	3	0.65 (0.55–0.77)	0.65 (0.53–0.80)			33%, *p* = 0.22
	≥75	3	0.88 (0.86–0.90)	0.81 (0.71–0.93)			78%, *p *< 0.01
	≥85*	1	0.94 (0.71–1.25)	0.94 (0.71–1.25)			NA
Region		8					
	North America	4	0.88 (0.86–0.90)	0.67 (0.48–0.92)	0.87 (0.85–0.89)	0.75 (0.66–0.85)	85%, *p *< 0.01
	Europe	2	0.85 (0.77–0.95)	0.84 (0.72–0.98)			37%, *p* = 0.18
	Asia	2	0.68 (0.60–0.76)	0.68 (0.60–0.76)			0%, *p* = 0.49
Diabetes proportion (%)		6					
	≥20	3	0.88 (0.86–0.90)	0.70 (0.53–0.93)	0.87 (0.85–0.89)	0.71 (0.58–0.86)	92%, *p *< 0.01
	<20	3	0.70 (0.59–0.83)	0.71 (0.56–0.91)			49%, *p* = 0.14
Hypertension proportion (%)		7					
	≥50	5	0.88 (0.86–0.90)	0.81 (0.73–0.91)	0.87 (0.85–0.89)	0.76 (0.67–0.87)	60%, *p* = 0.01
	<50	2	0.67 (0.59–0.75)	0.49 (0.23–1.08)			80%, *p* = 0.03
Follow-up (y)		7					
	≥7	3	0.74 (0.60–0.92)	0.66 (0.42–1.03)	0.88 (0.86–0.90)	0.76 (0.67–0.87)	71%, *p* = 0.03
	<7	4	0.88 (0.86–0.90)	0.78 (0.68–0.90)			73%, *p *< 0.01
Study design		8					
	Prospective study	3	0.74 (0.60–0.92)	0.66 (0.42–1.03)	0.87 (0.85–0.89)	0.75 (0.66–0.85)	71%, *p* = 0.03
	Retrospective study	4	0.88 (0.86–0.90)	0.78 (0.68–0.90)			73%, *p *< 0.01
	Nested case-control study	1	0.69 (0.61–0.77)	0.69 (0.61–0.77)			NA

*Ramos *et al*. [28] reported two groups about 85 in one study. †More than one groups about related data in one study. Abbreviations: HR, hazard ratio; 95% CI, 95% confidence interval; CHD/MI, coronary heart disease/myocardial infraction; CV events, cardiovascular events; NA, not available. Bold type, statistical significance.

### 3.4 Evidence grading and publication bias

**Fig. 4.**
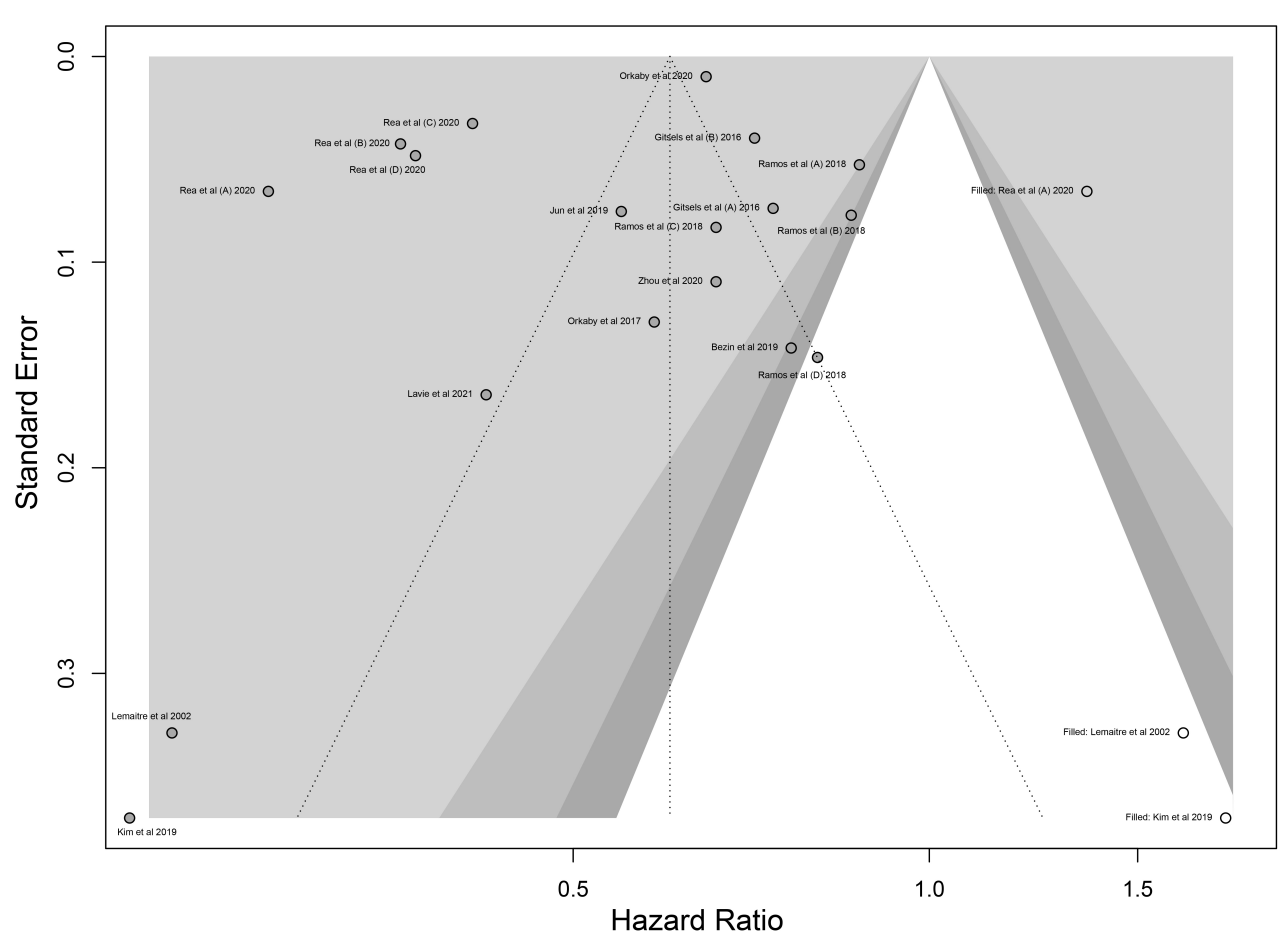
**Funnel plot with fill-and-trim method**. After trim-and-fill statistical process, the funnel plot seemed to be more symmetry.

According to the GRADE approach, evidence for all-cause mortality and CVD mortality was rated as “very low”, and for CHD/MI, stroke, total CV events, DM incidence, and cancer incidence was rated as “low”. Details have been given in Table 3. We analyzed potential publication bias for all-cause mortality, including most eligible studies (11 studies), and no evidence of publication bias was found (Egger’s test *p* = 0.246). The effect estimate of all-cause mortality was visualized and improved by “trim-and-fill” method. After the trim-and-fill statistical process, the revised funnel plot seemed to be more symmetry (Fig. 4).

**Table 3.** **GRADE assessment of quality of evidence**.

Outcomes	Risk of ${\mathrm{bias}}^{*}$	Inconsistency**	Indirectness	Imprecision†	Publication bias††	Large effect	Dose response	Residual bias	Quality of evidence‡
All-cause mortality	Not serious	Very serious	Not serious	Not serious	Undetected	Undetected	Undetected	Undetected	⊕ ○ ○ ○
									Very low
CVD mortality	Serious	Very serious	Not serious	Not serious	Not available	Undetected	Undetected	Undetected	⊕ ○ ○ ○
									Very low
CHD/MI	Serious	Serious	Not serious	Serious	Not available	Undetected	Undetected	Undetected	⊕ ⊕ ○ ○
									Low
Stroke	Serious	Serious	Not serious	Not serious	Not available	Undetected	Undetected	Undetected	⊕ ⊕ ○ ○
									Low
Total CV events	Serious	Serious	Not serious	Not serious	Not available	Undetected	Undetected	Undetected	⊕ ⊕ ○ ○
									Low
DM incidence	Serious	Not serious	Not serious	Serious	Not available	Undetected	Undetected	Undetected	⊕ ⊕ ○ ○
									Low
Cancer incidence	Serious	Not serious	Not serious	Not serious	Not available	Undetected	Undetected	Undetected	⊕ ⊕ ○ ○
									Low

*Risk of bias of included studies were assessed by study number, NOS and ROBINS-I tools. **Serious inconsistency indicated significant heterogeneity of 80% > *$I^{2}$* > 50%, *p* value < 0.05; very serious inconsistency indicated significant heterogeneity of *$I^{2}$* ≥ 80%, *p* value < 0.05. †Serious imprecision indicated the confidence intervals for pooled results were board (larger than 0.3). ††Publication bias was evaluated by Egger’s test, a *p*-value < 0.1 indicated significant publication bias (Detected bias). The analysis was performed for all-cause mortality since there were 11 studies included. ‡If there was one “serious”, the evidence was “low” and if there was one “Very serious”, the evidence was “Very low”. Abbreviation: CVD, cardiovascular disease; CHD/MI, coronary heart disease/myocardial infraction; DM, diabetes mellitus.

## 4. Discussion

By 2050, more than 45 million Americans will be 75 years or older, with a great proportional rate of 85 years and older people [3]. Evidence suggested that the incidence and prevalence of atherosclerotic cardiovascular disease (ASCVD) increases with age and keeps the leading cause of total mortality, disturbs the quality of life, and extends medical costs [9, 36]. Thus, proper management and care on those older populations are urgent. In our meta-analysis, it was found that statin use might be associated with a significant risk reduction on all-cause mortality, CVD mortality, CHD/MI, stroke and total CV events, and the reduced risks was 46%, 49%, 17%, 21% and 25%, respectively. Risk reduction in all-cause mortality keeps significant at higher ages regardless of diabetes as well as hypertension status. No significant association was found between statin use and diabetes incidence or cancer incidence. Briefly, there findings supported the positive correlation between statin use and CVD primary prevention in older population. Due to the observational nature, we still require further investigations to address the causality.

The beneficial role of statin use in all-cause mortality was consistent with the results from former clinical trials, and statin preserved risk role of elevated LDL-C in older people. A limitation of those trials was the limited sample size in subgroups of ≥80 and ≥85 years individuals. Current study conducted subgroup analyses on the even older populations (≥70 and ≥75 years people), and the abundant studies/sample size demonstrated a robust favorable role of statin use [37]. Primary prevention in older people coupled with DM needs more clinical evidence, and the role of statin use in these distinct people is still controversial. An analysis carried out on DM status indicated a negative association between statin use and all-cause mortality was only obvious in diabetic participants, which highlights the requirement for more use of statin therapy in older people with DM for the primary prevention [38]. The cohort study involved 5152 people aged from 66 to 96 years. It was showed that statins had significant association with reduced risk of all-cause mortality of diabetic individuals compared with non-DM individuals. Meanwhile, glucose-lowering therapy had no relationship with the all-cause mortality in diabetic individuals [38]. From epidemiological studies, we concluded that DM is always correlated with 2 to 4-fold higher total CV events risk, patients with long-standing DM (no less than 10 years) may have further CHD events [39, 40, 41]. Current study illustrates consistent results on all-cause mortality regardless of the diabetes status that is clinically plausible.

The aging people have a higher risk of drugs adverse events due to multiple comorbidities, polypharmacy, and altered pharmacokinetics and pharmacodynamics. The safety of statin in these people is a major of concern related to statin therapy continuation. Many older people are companied with hypertension especially in the high CVD risk populations. In current study, we revealed that statin use links to reduced risks of all-cause mortality regardless of the hypertension status, which implies that statin can be recommended to older people suffering from mild CVD (less proportion of hypertension). In a meta-analysis of more than 3 million older subjects, only 47.9% statin users were adherent to therapy after one year of follow-up [42]. According to a current study, there was no significant association between statin use and risk of DM or cancer incidence, and such results were in line with the conclusion from previous randomized controlled trials (RCTs) that investigated the primary prevention in older people [43, 44, 45]. However, evidence that focuses on general mixed populations (including both primary and secondary prevention) reported a 9% to 55% increased risk of diabetes in statin-use participants compared with the no users [46]. Another meta-analysis revealed that older statin-use participants were associated with 21% of decreased risk of T2DM compared with younger participants [47]. Based on these results, statin-associated DM risk will be more obvious in people with extremely high CVD risk such as extremely old people who have already suffered from serious CVD, metabolic syndrome etc. [46, 48]. Older people are always heterogeneous in many aspects (i.e., demographic characteristics, health and body function). Unfortunately, these confounders are not well elaborated in RCTs especially those with ≥75 years participants, and the clinical value can also be limited [12, 49]. Worse still, the follow-up period and the sample size are not abundant considering the limited life expectancy for older people included in RCTs. On the other hand, although our study is based on data from observational studies, the data are more generalizable with more available sample sizes, longer follow-up and mostly adjusted estimate size (HR).

When comparing with other similar studies, a recent meta-analysis incorporated 40 RCTs to investigate the efficacy and safety of statins for primary prevention of CVD with 94,283 patients at different ages [50]. That study displayed that statin use significantly reduced the risk of all-cause mortality (HR: 0.89, 95% CI: 0.85–0.93) in the included populations [50]. However, no further data about the elderly can be found. Another Bayesian analysis that calculated the available data on older people (>75 years) from 35 RCTs indicated that statin use for CVD primary prevention would have a significant lower mortality (*p* = 0.03) [51]. The beneficial role of statin use for the primary prevention was established, but it was not robust. Awad *et al*. [14] performed a meta-analysis on observational studies and they revealed that statin use was associated with reduced risk of all-cause mortality, CVD mortality and stroke, and no association was found for CHD/MI. Two more studies (including one study in 2021 and one study lacked) were included in current study, and there was a possibility for reduced CHD/MI with statin use (HR: 0.83, 95% CI: 0.69–1.00). Other outcomes became more confirming and robust, and HRs for them became smaller [33, 35]. The total CV events was one more primary outcome in current study over Awad *et al*. [14], and statin use kept negatively associated with total CV events (HR: 0.75, 95% CI: 0.66–0.85). As Awad *et al*. [14] stated, their findings on all-cause mortality need more caution when being applied for clinical practice, because the included older people with short life expectancy are less likely to receive statins, which can be outcome bias that was introduced into the observed results [14]. Current study included more sample size with additional eligible studies, and most final pooled results were robust, because the 95% CIs were far away from 1.00 and were confirmed by comprehensive sensitivity analysis and subgroup analysis. To avoid analysis bias or alternative approach, the study protocol has been successfully registered (INPLASY2021120045) before formally writing the manuscript. Both studies revealed that there was no association between DM and cancer incidence. In short, the cumulating evidence is widely consistent in general populations and has been validated through multiple subgroup analyses. To date, our study is one of the most powerful meta-analysis on this topic based on observational studies. However, we also acknowledge that the findings, especially the CHD/MI, need more evidence to confirm the robustness and promote the utility in clinical practice.

## 5. Limitation

Several limitations should be illustrated. Firstly, there is great heterogeneity among analyses on the primary outcomes, and the heterogeneity still exists in all-cause mortality by omitting high heterogeneous studies. We hypothesized that it might be caused by the inconsistent characteristics of older people in many aspects and the poor nature of observational studies. The results on CVD mortality, CHD/MI, stroke and total CV events are not significantly changed whether the studies of great heterogeneity were excluded or not. The second limitation is the poor quality of included observational studies whose average NOS was 6.67. The evidence on all-cause mortality and CVD mortality is evaluated as “very low”. Actually, there are only 4 high quality studies, final pooled results require more caution to be applied on clinical practice. Moreover, even though we found most of the results were robust, we performed sensitivity and subgroup analyses to try to find the source of heterogeneity. Thirdly, in terms of outcomes of interest, the definitions on CVD or CV events are various. We consistently pursue uniformed definitions on CVD and seek for individualized differences and commonalities among people, just as the guidelines’ requirement. It is suggested that further studies should be more precise on that. Finally, due to the nature of observational studies, we failed to draw strong causality, so we need to compare the results of meta-analysis based on observational studies and further RCTs with larger sample size and/or longer follow-up period. In that case, we will out forward more useful suggestions for the clinical duties and public health.

## 6. Conclusions

Statin use is useful for primary prevention for all-cause mortality, CVD mortality, CHD/MI, stroke and total CV events. The relevance keeps existing regardless of diabetes and hypertension status, and even older populations. Furthermore, no association was found for DM and cancer incidence. These findings supported that statin use is suitable for older people in primary prevention setting especially those with high CVD risk. Most importantly, considering the observational nature of evidence, more relevant trials should be conducted in older people.

## Supplementary material

Supplementary material associated with this article can be found, in the online version, at https://doi.org/10.31083/j.rcm2304114.

## Appendix

See details in **Appendix File 1**.
