# Supplementary material for: Statin use in older people primary prevention on cardiovascular disease: an updated systematic review and meta-analysis
Source: Rev Cardiovasc Med. 2022 Mar 24;23(4):114. doi: 10.31083/j.rcm2304114 (PMC11273788; doi:10.31083/j.rcm2304114)
Supplement: Supplementary file 1 [file 2153-8174-23-4-114-s1.doc]

Supplementary Table 1. PRISMA statements.

| **Section/topic** | **#** | **Checklist item** | **Reported on page #** |
| --- | --- | --- | --- |
| **TITLE** | | | 1 |
| Title | 1 | Identify the report as a systematic review, meta-analysis, or both. | 1 |
| **ABSTRACT** | | | 2 |
| Structured summary | 2 | Provide a structured summary including, as applicable: background; objectives; data sources; study eligibility criteria, participants, and interventions; study appraisal and synthesis methods; results; limitations; conclusions and implications of key findings; systematic review registration number. | 2 |
| **INTRODUCTION** | | | 3-4 |
| Rationale | 3 | Describe the rationale for the review in the context of what is already known. | 3 |
| Objectives | 4 | Provide an explicit statement of questions being addressed with reference to participants, interventions, comparisons, outcomes, and study design (PICOS). | 3-4 |
| **METHODS** | | | 4-7 |
| Protocol and registration | 5 | Indicate if a review protocol exists, if and where it can be accessed (e.g., Web address), and, if available, provide registration information including registration number. | 4 |
| Eligibility criteria | 6 | Specify study characteristics (e.g., PICOS, length of follow-up) and report characteristics (e.g., years considered, language, publication status) used as criteria for eligibility, giving rationale. | 4-5 |
| Information sources | 7 | Describe all information sources (e.g., databases with dates of coverage, contact with study authors to identify additional studies) in the search and date last searched. | 4 |
| Search | 8 | Present full electronic search strategy for at least one database, including any limits used, such that it could be repeated. | 4 |
| Study selection | 9 | State the process for selecting studies (i.e., screening, eligibility, included in systematic review, and, if applicable, included in the meta-analysis). | 4-5 |
| Data collection process | 10 | Describe method of data extraction from reports (e.g., piloted forms, independently, in duplicate) and any processes for obtaining and confirming data from investigators. | 5 |
| Data items | 11 | List and define all variables for which data were sought (e.g., PICOS, funding sources) and any assumptions and simplifications made. | 5 |
| Risk of bias in individual studies | 12 | Describe methods used for assessing risk of bias of individual studies (including specification of whether this was done at the study or outcome level), and how this information is to be used in any data synthesis. | 5-6 |
| Summary measures | 13 | State the principal summary measures (e.g., risk ratio, difference in means). | 6-7 |
| Synthesis of results | 14 | Describe the methods of handling data and combining results of studies, if done, including measures of consistency (e.g., I^2^) for each meta-analysis. | 6-7 |
| Risk of bias across studies | 15 | Specify any assessment of risk of bias that may affect the cumulative evidence (e.g., publication bias, selective reporting within studies). | 6-7 |
| Additional analyses | 16 | Describe methods of additional analyses (e.g., sensitivity or subgroup analyses, meta-regression), if done, indicating which were pre-specified. | 6-7 |
| **RESULTS** | | | 7-10 |
| Study selection | 17 | Give numbers of studies screened, assessed for eligibility, and included in the review, with reasons for exclusions at each stage, ideally with a flow diagram. | 7 |
| Study characteristics | 18 | For each study, present characteristics for which data were extracted (e.g., study size, PICOS, follow-up period) and provide the citations. | 7 |
| Risk of bias within studies | 19 | Present data on risk of bias of each study and, if available, any outcome level assessment (see item 12). | 7 |
| Results of individual studies | 20 | For all outcomes considered (benefits or harms), present, for each study: (a) simple summary data for each intervention group (b) effect estimates and confidence intervals, ideally with a forest plot. | 7 |
| Synthesis of results | 21 | Present results of each meta-analysis done, including confidence intervals and measures of consistency. | 8-10 |
| Risk of bias across studies | 22 | Present results of any assessment of risk of bias across studies (see Item 15). | 8-10 |
| Additional analysis | 23 | Give results of additional analyses, if done (e.g., sensitivity or subgroup analyses, meta-regression [see Item 16]). | 8-10 |
| **DISCUSSION** | | | 10-13 |
| Summary of evidence | 24 | Summarize the main findings including the strength of evidence for each main outcome; consider their relevance to key groups (e.g., healthcare providers, users, and policy makers). | 10-12 |
| Limitations | 25 | Discuss limitations at study and outcome level (e.g., risk of bias), and at review-level (e.g., incomplete retrieval of identified research, reporting bias). | 12-13 |
| Conclusions | 26 | Provide a general interpretation of the results in the context of other evidence, and implications for future research. | 13 |
| **FUNDING** | | | 18 |
| Funding | 27 | Describe sources of funding for the systematic review and other support (e.g., supply of data); role of funders for the systematic review. | 18 |

*From:*  Moher D, Liberati A, Tetzlaff J, Altman DG, The PRISMA Group (2009). Preferred Reporting Items for Systematic Reviews and Meta-Analyses: The PRISMA Statement. PLoS Med 6(7): e1000097. doi:10.1371/journal.pmed1000097

For more information, visit: **www.prisma-statement.org**.

Supplementary Table 2. Literature search strategy for relevant databases.

|  | **PubMed/Web of Science Search Strategy** |
| --- | --- |
| **1 Aging** | ("Aging"[Mesh] OR " Senescence" OR "Biological Aging" OR "Aging, Biological" OR "Elderly" OR "Aged" OR "Frail") |
| **2 Stain** | ("Stain" OR " atorvastatin " OR " fluvastatin" OR " lovastatin" OR " pitavastatin” OR "pravastatin" OR " rosuvastatin " OR " simvastatin " OR “lipitor” OR “zocor” OR “mevacor” OR “altoprev” OR “crestor” OR “lescol” OR “livalo” OR “Pravachol” OR “Hydroxymethylglutaryl-CoA Reductase Inhibitors”) |
| **3 Cardiovascular disease** | ("Cardiovascular disease" OR "Disease, cardiovascular" OR "Diseases, cardiovascular" OR "Coronary disease" OR "Coronary heart disease" OR "Disease coronary heart” OR “Myocardial infarction” OR “Infarct, myocardial” OR “Heart Attack” OR “Heart attacks” OR “Stroke” OR “Cerebrovascular accident” OR “Brain vascular accident” OR “Cerebral stroke” OR “Acute cerebrovascular accident” OR “Apoplexy”) |
| **4 Observational study** | (Observational study[pt] OR Prospective study[pt] OR retrospective study[tiab] OR cohort study[tiab] OR case-control study[mesh:noexp] OR Nested case-control study [tiab] OR cross-sectional study[ti]) |
| **5** | (animals[mh] NOT humans [mh]) |
| **6** | 1 AND 2 AND 3 AND 4 |
| **7** | 6 NOT 5 |

Search strategy in PubMed/Web of Science as example.

Supplementary Table 3. Confounders of adjustment in included studies.

| **Study** | **Confounders** |
| --- | --- |
| Lemaitre et al^1^ (2002) | sex, age, diabetes, prevalent CVD (angina, coronary bypass surgery, angioplasty, carotid endarterectomy, bypass procedure on a leg artery) |
| Alperovitch et al^2^ (2015) | sex, center, diabetes, BMI, alcohol consumption, smoking, hypertension, cardiac rhythm disorder, antithrombotic therapy, triglycerides, low density lipoprotein/high density lipoprotein ratio |
| Gitsels et al^3^ (2016) | sex, age, year of birth, socioeconomic status, diabetes, hypercholesterolaemia, blood pressure, regulating drugs, BMI, smoking status, general practice. |
| Orkaby et al^4^ (2017) | age, race, BMI, comorbidities, life-style factors, concurrent medications |
| Ramos et al^5^ (2018) | age, sex, height, weight, systolic and diastolic BP, Medea deprivation index, smoking, glucose, total cholesterol, HDL and LDL, obesity, hypercholesterolemia, valvular heart disease, atrial fibrillation, benign neoplasm, hypertension, asthma, chronic obstructive pulmonary disease, sleep apnea, arthritis, hyperthyroidism, hypothyroidism, chronic kidney disease, and treatments other than statins (diuretics, beta blocking agents, calcium channel blockers, agents acting on the renin-angiotensin system, other antihypertensives, antidiabetic drugs, corticosteroids for systemic use, antiinflammatory and antirheumatic drugs, psycholeptics, and psychoanaleptics). |
| Bezin et al^6^ (2019) | time-dependent confounders and high-dimensional propensity score for statin initiation |
| Jun et al^7^ (2019) | age, sex, income category,T2DM, hypertension, previous use of other lipid-lowering drugs (fibrates or ezetimibe). |
| Kim et al^8^ (2019) | age, sex, hypertension, DM, chronic thyroid disease, chronic pulmonary disease, congestive heart failure, atrial fibrillation, renal insufficiency (estimated glomerular filtration rate<60 mL/min/1.73m2), body mass index, baseline lipid profiles, and current medications (anticoagulant, antiplatelet, and number of antihypertensive agents). |
| Orkaby et al^9^ (2020) | age, sex, race, ethnicity, BMI, region of country, aging-specific variables, smoking status |
| Rea et al^10^ (2020) | age, sex, statin at cohort entry, other drugs, previous hospitalizations |
| Zhou et al^11^ (2020) | age, sex, smoking status, current drinkers, BMI, DM, Hypertension, previous medication |
| Lavie et al^12^ (2021) | age, sex, socioeconomic status, immigration status, systolic and diastolic BP, smoking status, BMI, blood glucose concentration (mg/dL), total cholesterol, LDL cholesterol, HDL cholesterol, triglycerides, CCI andmedication use: calcium channel blockers, use of beta-blockers, thiazides, oral steroids, oral contraceptives, antipsychotics, phenytoin, thyroxine, and fibrates |

Abbreviations: CVD, cardiovascular disease; BMI, body mass index; HDL, high density lipoprotein; LDL, low density lipoprotein; T2DM, type 2 diabetes mellitus; BP, blood pressure; CCI: Charlson Comorbidity Index

Supplementary Table 4. Quality assessment of the included studies.

|  | **Study** | **Selection** | | | | **Comparability** | **Outcome** | | | **Total score** |
| --- | --- | --- | --- | --- | --- | --- | --- | --- | --- | --- |
|  |  | Exposed cohort | Nonexposed cohort | Ascertainment of exposure | Outcome of interest |  | Assessment of outcome | Length of follow-up | Adequacy of follow-up |  |
| **2002** | Lemaitre et al^1^, | * | * | * | * | * | * | * | * | 8 |
| **2015** | Alperovitch et al^2^, | * | * | * | * | ** | * | * | * | 9 |
| **2016** | Gitsels et al^3^, | * | * | * |  | * |  | * | * | 6 |
| **2017** | Orkaby et al^4^, | * | * | * | * | * | * | * |  | 7 |
| **2018** | Ramos et al^5^, | * | * | * | * | ** | * | * |  | 8 |
| **2019** | Bezin et al^6^, | * | * |  |  |  | * | * |  | 4 |
| **2019** | Jun et al^7^, | * | * |  | * | * | * |  |  | 5 |
| **2019** | Kim et al^8^, | * | * |  | * | * | * | * |  | 6 |
| **2020** | Orkaby et al^9^, | * | * |  | * | ** | * | * | * | 8 |
| **2020** | Rea et al^10^, | * | * |  |  | * | * | * | * | 6 |
| **2020** | Zhou et al^11^, | * | * |  | * | ** | * | * |  | 7 |
| **2021** | Lavie et al^12^, | * | * |  | * | * | * | * |  | 6 |

Supplementary Table 5. Study quality evaluation using Risk of Bias in Non-randomized Studies of Interventions (ROBINS-I) Tool.

| **Study** | **Bias due to confounding** | **Bias in selection of participants into the study** | **Bias in classification of interventions** | **Bias due to deviations from intended interventions** | **Bias due to missing data** | **Bias in measurement of outcomes** | **Bias in selection of the reported result** | **Overall bias** |
| --- | --- | --- | --- | --- | --- | --- | --- | --- |
| Lemaitre et al^1^ (2002) | Serious | Serious | Low | Low | Low | Moderate | Low | Serious |
| Alperovitch et al^2^ (2015) | Moderate | Serious | Low | Low | Low | Moderate | Low | Serious |
| Gitsels et al^3^ (2016) | Moderate | Serious | Low | Low | Low | Low | Low | Serious |
| Orkaby et al^4^ (2017) | Serious | Serious | Moderate | Low | Low | Moderate | Low | Serious |
| Ramos et al^5^ (2018) | Moderate | Low | Low | Low | Low | Low | Low | Moderate |
| Bezin et al^6^ (2019) | Moderate | Low | Low | Low | Low | Low | Low | Moderate |
| Jun et al^7^ (2019) | Serious | Serious | Moderate | Low | Low | Low | Low | Serious |
| Kim et al^8^ (2019) | Moderate | Low | Low | Low | Low | Moderate | Low | Moderate |
| Orkaby et al^9^ (2020) | Moderate | Low | Low | Low | Low | Low | Low | Moderate |
| Rea et al^10^ (2020) | Low | Low | Low | Low | Moderate | Moderate | Moderate | Moderate |
| Zhou et al^11^ (2020) | Moderate | Serious | Low | Low | Low | Low | Low | Serious |
| Lavie et al^12^ (2021) | Serious | Low | Low | Low | Low | Moderate | Moderate | Serious |

Supplementary material 1. Detailed definitions on the outcomes of interest.

The definitions of study outcomes^13-16^

**All-cause mortality**

The all-cause mortality included all deaths were confirmed with two independent sources (e.g. family, general practitioner/family physician or public death notice). Although not an ASPREE endpoint, trajectory to death was also collected according to the following categories including (1) cancer related death; (2) dementia related death; (3) coronary heart disease death (i.e. myocardial infarction, sudden cardiac death, rapid cardiac death, cardiac failure with coronary cause, other coronary death); (4) non‐coronary vascular death (e.g. abdominal aortic aneurysm rupture, cardiomyopathy, or myocarditis); (5) stroke death; (6) major haemorrhage death; or (7) death from other cause.

**CVD mortality**

Known as fatal CVD, defined as any death in which the underlying cause was coronary heart disease, stroke (details see ‘stroke’ definition) or other CVD event.

**CHD/MI**

CHD/MI includes both fatal and nonfatal CHD/MI and is defined as any cardiac event demonstrating typical rise in biochemical markers of myocardial necrosis (*i.e.* troponin or CK‐MB) with at least one of: (1) ischemic symptoms; development of pathologic Q waves on the ECG; (2) ECG changes indicative of ischemia (ST segment elevation or depression) or; (3) coronary artery intervention (*e.g.* coronary angioplasty) as per the American College of Cardiology & European Society of Cardiology definition. Development of proven new pathologic Q waves on serial ECGs, or evidence of acute, healed or healing MI on autopsy was also be considered an MI endpoint.

**Stroke**

Stroke is defined as per World Health Organization (WHO) criteria as ‘rapidly developing clinical signs of focal (or global) disturbance of cerebral function lasting more than 24 hours (unless interrupted by surgery or death) with no apparent cause other than of vascular origin'. Stroke endpoint included all fatal/nonfatal stroke types recorded in the ASPREE study: hemorrhagic stroke, ischemic stroke, ischemic stroke with hemorrhagic transformation, sub-arachnoid hemorrhage stroke, and stroke type uncertain.

**Total CV events**

Known as a composite outcome of nonfatal myocardial infraction, fatal/nonfatal stroke (more details see the “stroke” definition), or coronary heart disease death etc.

**DM incidence**

Known as the onset of new diabetes events, not restricted on the diabetes types.

**Cancer incidence**

Known as the onset of new cancer events, not restricted on special cancer types.

**References**

[1] Lemaitre RN, Psaty BM, Heckbert SR, Kronmal RA, Newman AB, Burke GL. Therapy with Hydroxymethylglutaryl Coenzyme a Reductase Inhibitors (Statins) and Associated Risk of Incident Cardiovascular Events in Older Adults: evidence from the Cardiovascular Health Study. Archives of Internal Medicine. 2002; 162: 1395.

[2] Alpérovitch A, Kurth T, Bertrand M, Ancelin M, Helmer C, Debette S, *et al*. Primary prevention with lipid lowering drugs and long term risk of vascular events in older people: population based cohort study. British Medical Journal. 2015; 350: h2335.

[3] Gitsels LA, Kulinskaya E, Steel N. Survival Benefits of Statins for Primary Prevention: a Cohort Study. PLoS ONE. 2016; 11: e0166847.

[4] Orkaby AR, Gaziano JM, Djousse L, Driver JA. Statins for Primary Prevention of Cardiovascular Events and Mortality in Older Men. Journal of the American Geriatrics Society. 2017; 65: 2362–2368.

[5] Ramos R, Comas-Cufí M, Martí-Lluch R, Balló E, Ponjoan A, Alves-Cabratosa L*, et al.* Statins for primary prevention of cardiovascular events and mortality in old and very old adults with and without type 2 diabetes: retrospective cohort study. British Medical Journal*.* 2018; 362: k3359.

[6] Bezin J, Moore N, Mansiaux Y, Steg PG, Pariente A. Real-Life Benefits of Statins for Cardiovascular Prevention in Elderly Subjects: a Population-Based Cohort Study. The American Journal of Medicine. 2019; 132: 740–748.e7.

[7] Jun JE, Cho I, Han K, Jeong I, Ahn KJ, Chung HY, *et al*. Statins for primary prevention in adults aged 75 years and older: a nationwide population-based case-control study. Atherosclerosis. 2019; 283: 28–34.

[8] Kim K, Lee CJ, Shim C, Kim J, Kim B, Park S, *et al*. Statin and clinical outcomes of primary prevention in individuals aged >75 years: the SCOPE-75 study. Atherosclerosis. 2019; 284: 31–36.

[9] Orkaby AR, Driver JA, Ho Y, Lu B, Costa L, Honerlaw J, *et al*. Association of Statin Use with all-Cause and Cardiovascular Mortality in us Veterans 75 Years and Older. The Journal of the American Medical Association. 2020; 324: 68.

[10] Rea F, Mancia G, Corrao G. Statin treatment reduces the risk of death among elderly frail patients: evidence from a large population-based cohort. Eur J Prev Cardiol*.* 2020. (in press)

[11] Zhou Z, Ofori-Asenso R, Curtis AJ, Breslin M, Wolfe R, McNeil JJ, *et al*. Association of Statin Use with Disability-Free Survival and Cardiovascular Disease among Healthy Older Adults. Journal of the American College of Cardiology. 2020; 76: 17–27.

[12] Lavie G, Hoshen M, Leibowitz M, Benis A, Akriv A, Balicer R, *et al*. Statin Therapy for Primary Prevention in the Elderly and its Association with New-Onset Diabetes, Cardiovascular Events, and all-Cause Mortality. The American Journal of Medicine. 2021; 134: 643–652.

[13]. McNeil JJ, Nelson MR, Woods RL, et al. Effect of Aspirin on All-Cause Mortality in the Healthy Elderly. *N Engl J Med* 2018; **379**(16): 1519-28.

[14]. McNeil JJ, Woods RL, Nelson MR, et al. Effect of Aspirin on Disability-free Survival in the Healthy Elderly. *N Engl J Med* 2018; **379**(16): 1499-508.

[15]. Ruiz-Canela M, Hruby A, Clish CB, Liang L, Martínez-González MA, Hu FB. Comprehensive Metabolomic Profiling and Incident Cardiovascular Disease: A Systematic Review. *J Am Heart Assoc* 2017; **6**(10).

[16]. Owolabi MO, Thrift AG, Mahal A, et al. Primary stroke prevention worldwide: translating evidence into action. *Lancet Public Health* 2021.
