# Supplementary material for: Statin use in older people primary prevention on cardiovascular disease: an updated systematic review and meta-analysis
Source: Rev Cardiovasc Med. 2022 Mar 24;23(4):114. doi: 10.31083/j.rcm2304114 (PMC11273788; doi:10.31083/j.rcm2304114)
Supplement: Supplementary file 2 [file 2153-8174-23-4-114-a1.pdf]

International Platform of Registered Systematic Review and Meta-analysis Protocols

INPLASY PROTOCOL

To cite: Huang et al. Statin use in older people primary prevention on cardiovascular disease: an updated systematic review and meta-analysis. Inplasy protocol 2021120045. doi: 10.37766/inplasy2021.12.0045

Received: 08 December 2021

Published: 08 December 2021

Corresponding author:
Ru Ya

445291614@qq.com

Author Affiliation:
Shanghai Third Rehabilitation Hospital.

Support: **No financial supportNo fininfi.**

Review Stage at time of this submission: **Data analysis.**

Conflicts of interest:
None declared.

Statin use in older people primary prevention on cardiovascular disease: an updated systematic review and meta-analysis

Huang, H[1]; Zhu, H[2]; Ya, R[3].

Review question / Objective: Statin use in older people for the primary prevention for cardiovascular disease is still controversial. We conducted this systematic review and meta-analysis to (1) investigate the cardiovascular disease (CVD) primary prevention via statin use in older populations; (2) make updated clinical advice to high CVD risk populations.Statin use in older people for the primary prevention for cardiovascular disease is still contyroversial.
Condition being studied: There have been numerous observational studies and randomized controlled trials (RCTs) on such topic. Therefore, we desire to perform a pooled analyses with high quality studies.

INPLASY registration number: This protocol was registered with the International Platform of Registered Systematic Review and Meta-Analysis Protocols (INPLASY) on 08 December 2021 and was last updated on 08 December 2021 (registration number INPLASY2021120045).

INTRODUCTION

Review question / Objective: Statin use in older people for the primary prevention for cardiovascular disease is still controversial. We conducted this systematic review and meta-analysis to (1) investigate the cardiovascular disease (CVD) primary prevention via statin use in older populations; (2) make updated clinical advice to high CVD risk populations.Statin use in older people for the primary prevention for cardiovascular disease is still contyroversial.

Rationale: It is well-established that statin use is recommended for secondary prevention on CVD in people older people

Huang et al. Inplasy protocol 2021120045. doi:10.37766/inplasy2021.12.0045 Downloaded from https://inplasy.com/inplasy-2021-12-0045/

as level A evidence, however, considerable evidence for primary prevention is lacking. Previous studies on this topic are not detailed, and results on the primary prevention for older people mostly come from small subgroup analyses. Therefore, we reasonably conduct this meta-analysis on intended topic.

Condition being studied: There have been numerous observational studies and randomized controlled trials (RCTs) on such topic. Therefore, we desire to perform a pooled analyses with high quality studies.

METHODS

Search strategy: We reviewed Pubmed, EMBASE, Cochrane Library and Web of Science for related literature from the inception to Sep-15-2021. We used a combination of relevant keywords and Medical Subject Headings (MeSH) terms going as: "Aging", "Aged", "elderly", "Statin", "atorvastatin", "cardiovascular disease", "cardiovascular events", "coronary heart disease", "myocardial infarction", "stroke" and "observational study". No restrictions were applied on language. Reference lists of the retrieved literature were also searched manually.

Participant or population: Being limited to or included a subgroup of older people aged ≥ 65 years using statin for primary prevention. No further restrictions on additional individual-level characteristics (e.g., sex, ethnicity, nation).

Intervention: Statin (atorvastatin, fluvastatin, lovastatin, pitavastatin, pravastatin, rosuvastatin, or simvastatin) use vs. no statin use for primary prevention.

Comparator: Statin (atorvastatin, fluvastatin, lovastatin, pitavastatin, pravastatin, rosuvastatin, or simvastatin) use vs. no statin use for primary prevention.

Study designs to be included: Observational study.

Eligibility criteria: Articles were screened in two-step methods. They were initially screened for titles and abstracts, then the full texts of potentially eligible studies were reviewed by two authors. Any disagreements were resolved by the opinion from a discussion in a group panel with another author who is exceptional in cardiology and evidence-based medicine.The eligible criteria following PICOS principles were as follows: Populations: Being limited to or included a subgroup of older people aged ≥ 65 years using statin for primary prevention. No further restrictions on additional individual-level characteristics (e.g., sex, ethnicity, nation). Intervention/comparison: Statin (atorvastatin, fluvastatin, lovastatin, pitavastatin, pravastatin, rosuvastatin, or simvastatin) use vs. no statin use for primary prevention.Outcomes: At least one of the following outcomes: All-cause mortality, CVD mortality, CHD/MI, stroke or total CV events.Study design: Observational study. Only the most informative studies with longer follow-up could be included to avoid duplication. Clinical trials, reviews, case reports, conference abstracts and experimental studies were excluded. Studies without essential data are also excluded.

Information sources: We reviewed Pubmed, EMBASE, Cochrane Library and Web of Science for related literature from the inception to Sep-15-2021. We used a combination of relevant keywords and Medical Subject Headings (MeSH) terms going as: "Aging", "Aged", "elderly", "Statin", "atorvastatin", "cardiovascular disease", "cardiovascular events", "coronary heart disease", "myocardial infarction", "stroke" and "observational study". No restrictions were applied on language. Reference lists of the retrieved literature were also searched manually.

Main outcome(s): The primary outcomes included risk of all-cause mortality, CVD mortality, CHD/MI, stroke and total CV events.

Huang et al. Inplasy protocol 2021120045. doi:10.37766/inplasy2021.12.0045 Downloaded from https://inplasy.com/inplasy-2021-12-0045/

Additional outcome(s): The secondary outcomes included risk on no diabetes mellitus (NODM) and cancer incidence.

Data management: All data extracted from the primary studies were set in standardized tables.

Quality assessment / Risk of bias analysis: To evaluate the quality of included studies, we applied the Newcastle-Ottawa Scale (NOS) as previously, which has been validated for assessing the quality of nonrandomized controlled trials in meta-analyses. As for a 0-10 scale, each study was categorized as low (0-5), medium (6-7), of high (8-10) quality. Two authors performed a quality assessment on all of the included studies based on the method. In case of any disagreements, there would be a discussion between the two authors.

Strategy of data synthesis: Multivariable hazard ratio (HR) and the corresponding 95% confidence intervals (95% CIs) for outcome of interests obtained from Cox-Hazard regression analysis were mainly estimated with DerSimonian-Laird (D-L) random effects model because the assumptions involved accounted for the presence of within-study and between-study heterogeneity. Both results on the random-effects and fixed-effects were shown in the forest plots. The adjusted relative risk (RR) and odd ratio (OR) in primary studies were approximately considered HR. Fully adjusted HRs and standard errors (SEs) originating from the correspondence 95% CIs were logarithmically transformed to stabilized variance, and the distribution was normalized.

Subgroup analysis: XWe would also conduct post subgroup analyses to ascertain the influence of other design and individual factors as follows: different categories on age, region, diabetic characteristics, hypertension status, study follow-up period and study design.

Sensitivity analysis: A sensitivity analysis was performed by moving one study each turn to try to elaborate the causes of the heterogeneity in each outcome of interest.

Language: English.

Country(ies) involved: China.

Keywords: Statin, Older people, Cardiovascular disease, Primary prevention, meta-analysis.

Contributions of each author:
Author 1 - Hao Huang - Study design, paper writing and statistical analysis.
Email: huanghaosankang@126.com
Author 2 - Hechen Zhu.
Email: zhuhechenhuashan@126.com
Author 3 - Ru Ya.
Email: yarusankang@126.com

Huang et al. Inplasy protocol 2021120045. doi:10.37766/inplasy2021.12.0045 Downloaded from https://inplasy.com/inplasy-2021-12-0045/
